# Plasma Scalpels: Devices, Diagnostics, and Applications

**DOI:** 10.3390/biomedicines10112967

**Published:** 2022-11-17

**Authors:** Ao Xiao, Dawei Liu, Dongcheng He, Xinpei Lu, Kostya (Ken) Ostrikov

**Affiliations:** 1 State Key Lab of Advanced Electromagnetic Engineering and Technology, School of Electrical and Electronic Engineering, Huazhong University of Science and Technology, Wuhan 430074, China; 2 Wuhan National High Magnetic Field Center, Wuhan 430074, China; 3 Jiangsu Bonss Medical Technology Co., Ltd., Taizhou 225316, China; 4 School of Chemistry and Physics, Queensland University of Technology (QUT), Brisbane, QLD 4000, Australia; 5 Centre for Materials Science, Queensland University of Technology (QUT), Brisbane, QLD 4000, Australia

**Keywords:** plasma scalpel, argon plasma coagulation, discharge in saline, diagnostics, surgery

## Abstract

The plasma scalpel is an application of gas discharges in electrosurgery. This paper introduces the device structure and physicochemical parameters of the two types of plasma scalpels, namely, a single-electrode Ar discharge device (argon plasma coagulation) and a two-electrode discharge device in normal saline. The diagnostic methods, including the voltage and current characteristics, optical emission spectroscopy, electron spin resonance, and high-speed imaging, are introduced to determine the critical process parameters, such as the plasma power, the gas temperature, the electron density, and the density of active species, and study the ignition dynamics of the plasma discharges in water. The efficacy of the plasma scalpel is mainly based on the physical effects of the electric current and electric field, in addition to the chemical effects of high-density energetic electrons and reactive species. These two effects can be adjusted separately to increase the treatment efficacy of the plasma scalpel. Specific guidance on further improvements of the plasma scalpel devices is also provided.

## 1. Introduction

A plasma scalpel is an electrosurgical medical device that uses gas discharge for tissue treatment. It mainly generates a spark discharge between the probe electrode and the target tissue, and produces a surface thermal effect on the biological tissue in a non-contact manner, thereby realizing cutting and ablation. The plasma scalpel was first introduced in open surgery in the late 1970s [1] and in endoscopy in 1991 [2,3]. The device can be easily inserted into cavities and areas of the patient’s body for minimally invasive surgery, and, currently, it has become one of the most commonly used endoscopic coagulation techniques [4].

In the following sections, we will introduce the types, device structures, physicochemical parameters, and diagnostic methods of the plasma scalpel, biological tissue responses to electrical discharges, and discuss the plasma scalpel’s recent developments and clinical applications, followed by the outlook on the development of plasma scalpels.

## 2. Types and Structures of Plasma Scalpels

Plasma scalpels are mainly divided into two categories: in one, the plasma is generated on the power electrode with argon as a working gas, and its ground electrode is at the distal end; and in the other, the anode and ground electrodes are immersed in a low dielectric constant fluid, and the plasma is generated in a thin layer of water vapor near the anode.

**Figure 1:**
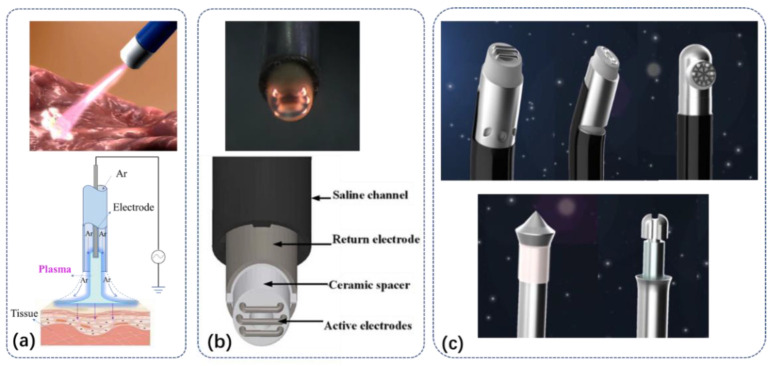
The structure and working diagram of a typical plasma scalpel. (**a**) Argon plasma coagulation (APC) with one power electrode inside the argon flow tube and the other distal ground electrode. (**b**) The plasma scalpel with the power and ground electrode inside the operation handle generates plasma in the saline. (**c**) Various plasma scalpels manufactured by Jiangsu Bonss Medical Technology Co., Ltd. (Taizhou, China).

Argon single-electrode discharge technology, referred to as argon plasma coagulation (APC), mainly coagulates biological tissue by argon discharge under atmospheric pressure [3]. Argon is chosen for its relatively low cost, low breakdown voltage, and biochemical inertness. As shown in Figure 1a, the diameter of the discharge electrode is a few tenths of a millimeter of metal with a needle-like or spatula-like tip [5,6,7]. The ground electrode at the far end is connected to the human body. During the discharge treatment, the distance between the power electrode and the human tissue is 0.2–1 cm, and argon flows through the dielectric tube with the power electrode inside. The AC voltage amplitude of the plasma discharge is 4 kV, the frequency is 350 kHz, and the peak current after a gas breakdown is 2–5 A. Unlike traditional coagulation techniques where electrodes are in contact with tissue, the APC can ensure that coagulated tissue does not stick to the electrodes, and due to the increased electrical impedance of human tissue during coagulation, the point of contact between the plasma and tissue is easily moved to the point of bleeding, the unsolidified area, so that the depth of action of the solidified site is controlled within a few millimeters.

A typical two-electrode discharge device in normal saline is shown in Figure 1b [8], where the anode is a set of three metal-alloy wire electrodes mounted on the scalpel head [9,10,11], and the exposed stainless-steel tube is the return ground electrode. The anode is embedded in a ceramic spacer to ensure separation from the return ground electrode. The outer rubber tube acts as a channel for delivering physiological saline, establishing an electrical circuit from the anode to the ground electrode. The applied voltage is a high-pulsed voltage of 100 kHz frequency, and the amplitude is 140–230 V. The plasma is generated in a thin layer of water vapor near the anode; in addition, the anodes that generate the plasma include multi-needle electrodes, ring electrodes, and single flat electrodes [8] as shown in Figure 1c.

## 3. Physicochemical Characteristics of Plasma Scalpel

### 3.1. Diagnostics on Physicochemical Characteristics of APC

Due to the plasma’s small size and high electron density, the traditional invasive electrical probes, such as the Langmuir probe, cannot be applied to the diagnosis of plasma properties of APCs. The electron density and electric field of the APC plasma can be determined by the numerical simulations and optical emission spectroscopy (OES) measurements of the absolute light intensity emitted by nitrogen molecules. The emission spectrum of molecular nitrogen at atmospheric pressure consists of two-electron systems, namely N_2_(C-B) (“second positive system”) (11.05 eV) and N_2_^+^(B-X) (“first negative system”) (18.74 eV), which have two different high-energy states. This large energy difference results in the emission spectra of nitrogen molecules being sensitive to changes in the electron energy distribution function in nitrogen-containing plasmas; therefore, combining OES measurements and the collisional radiation model of nitrogen molecules can be used to infer information, such as the electron energy distribution and electric field [12].

The voltage-current measurement, photomicrography, and other means are combined to measure the process of APC treatment of a pig kidney with a discharge power of 30 W. The results showed that the APC discharge started with the spark discharge stage because the streamer propagated from the anode to the tissue during the positive period of the applied high voltage. After the spark discharge, the APC discharge entered the glow discharge stage, the electron density of the plasma is 9.9 × 10^21^/m^3^, the average electron kinetic energy is 2.6 eV, the electric field is 12 Td, and the gas temperature is 1400 K [13]. During the negative period, the glow discharge continued. Within 3 s, the APC generated a white–yellow coagulation area with a bottom radius of 3.2 mm and a depth of 1.6 mm on the surface of the pig kidney. Importantly, the tissue in this coagulation area was not carbonized or gasified [13].

The gas temperature of the plasma discharge can be decreased by lowering the input energy. The highly flexible non-thermal APC discharge can be used to treat precancerous and cancerous lesions, such as low- or high-grade cervical intraepithelial neoplasia and other gynecological tumors [14]. Electron spin resonance (ESR) is used to measure the amount and distribution of reactive species during non-thermal APC treatment of aqueous solutions and human foreskin tissue. The results showed that the amount of free radical species was significantly increased. The spin densities of 5,5-dimethylpyrroline N-oxide (DMPO)-OH and DPMO-H were higher compared to other radical signals in the non-thermal APC-treated solution. A large amount of carbon-centered radical DPMO-R was generated in the non-thermal APC-treated human foreskin tissue.

### 3.2. Diagnostics of Plasma Characteristics of Two-Electrode Plasma Scalpel

**Figure 2:**
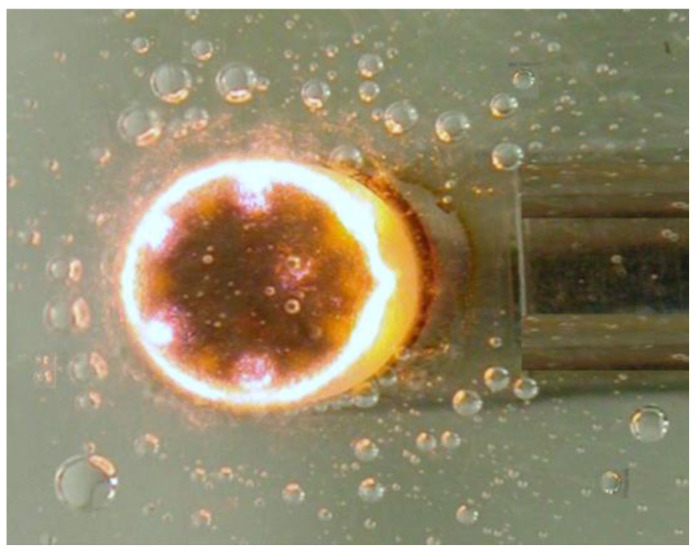
Plasma is generated in the water-vapor layer on the powered electrode.

The two-electrode plasma scalpel is discharged by immersing the anode and ground electrodes in a fluid with a low dielectric constant (Figure 1b) [15]. The liquid required during the plasma scalpel treatment is usually isotonic saline [11,16,17], containing 0.9% NaCl dissolved in water and other small amounts of buffer salts to maintain the optimum acid-base balance. Isotonic saline is conductive due to dissolved ions. When the local ohmic heating of the brine due to the electric field and current density near the anode exceeds the brine’s vaporization heat and heat dissipation rate, there is localized vaporization, and many tiny bubbles are observed emanating from the vicinity of the electrode (Figure 2). When the voltage is high enough [18], a thin water-vapor layer forms on the high field region of the electrode surface.

The formation of a vapor layer is a necessary condition for plasma generation. The molecular number density N in the vapor is significantly reduced compared with that in the liquid state. At the same time, due to the thin thickness of the vapor layer and the high conductivity of the brine, the electric field strength in the thin vapor layer increases rapidly, resulting in a locally higher E/N ratio, so that the gas breakdown generates a transient non-equilibrium glow discharge around the electrode. Depending on the applied voltage and electrode geometry, the discharge current is typically between 10 and 150 mA [19]. From the breakdown conditions of E/N = 100 Td in the water-vapor layer, the electron temperature of the plasma is 4–6 eV [19]. 

**Figure 3:**
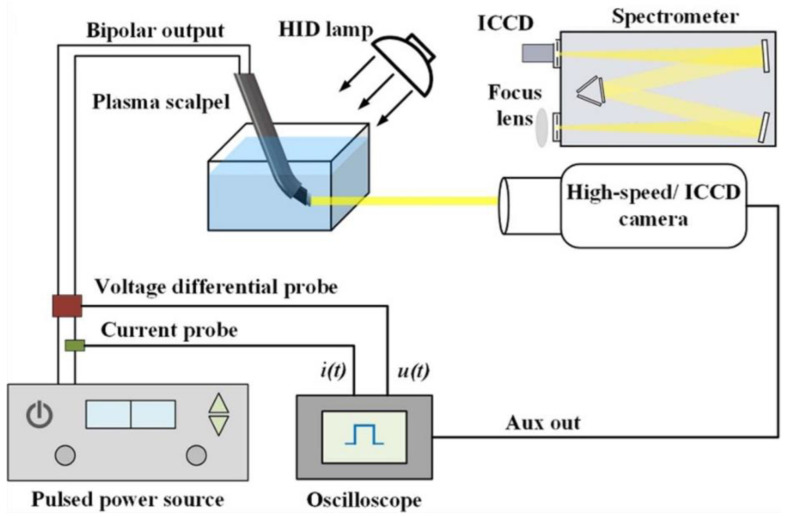
Schematic of the plasma diagnostics system for plasma scalpel Reproduced with permission from Ref. [8]. Copyright (2020) IOP Publishing, Ltd.

Optical emission spectroscopy (OES) and high-speed imaging are important diagnostic methods for discharge in water [20,21]. Figure 3 shows the diagnostic system of the plasma scalpel, which includes a high-voltage probe and a current probe to measure the electrical properties of the plasma [22,23,24], a high-speed camera to observe the vapor formation process, and a spectrometer and an ICCD camera to obtain the optical emissions spectra from the plasma discharge [8].

**Figure 4:**
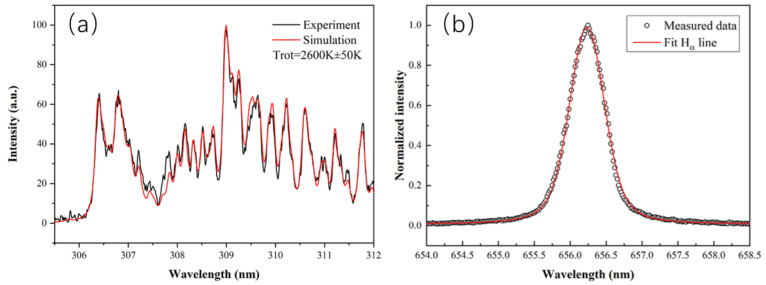
(**a**) OES of the plasma scalpel from 305.5–312 nm. The simulation of spectra by LIFBASE is also shown. (**b**) Voigt fitting of Hα line measured in the plasma. The fitting FWHM of Stark broadening is 0.208 nm, and the calculated electron density is 7.1 × 10^15^ cm^−3^. Reproduced with permission from Ref. [8]. Copyright (2020) IOP Publishing, Ltd.

In the OES of the water plasma, hydroxyl radical OH(A-X) at 309 nm and excited hydrogen atoms (the Balmer lines H_α_) at 626 nm can be observed. The emission band of OH (X^2^Π → A^2^Σ) around 309 nm is used to estimate the plasma gas temperature [25,26]. Figure 4a shows the emission spectrum of the plasma from 305.5 nm to 312 nm [27,28] and a simulated OH spectrum with a rotational temperature of 2600 K (calculated by LIFBASE). The matching between the measured and simulated spectra shows that the plasma gas temperature is estimated to be 2600 K [8]. This temperature is a lot higher than the vaporization temperature of moisture (100 °C), so the plasma in the water is able to maintain the vapor state around the electrodes. The electron density of the plasma can be obtained by Stark broadening of the Hα line [29,30]. Figure 4b below shows that the fitting FWHM of Stark broadening is obtained with a value of 0.208 nm, so the calculated electron density is 7.1 × 10^15^ cm^−3^ [8]; such a high electron density favors the production of reactive species in the plasma.

**Figure 5:**
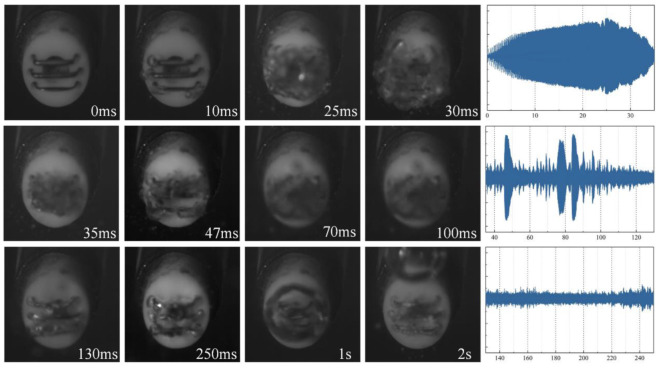
Vaporization dynamics of the plasma scalpel. On the right is the current waveform of three stages. Reproduced with permission from Ref. [8]. Copyright (2020) IOP Publishing, Ltd.

The working process of the plasma scalpel is divided into three stages: heating, evaporation, and discharge. The plasma scalpel’s current characteristics (Figure 5) also exhibit three discharge stage. These stages are recorded by high-speed cameras [8] and the plasma scalpel’s current characteristics [20]. As shown in Figure 5, in the first stage (0–35 ms), after many tiny bubbles form at the electrode corners, the size and number of bubbles increase with the increase in the voltage peak. When the bubbles completely cover the electrode, the current amplitude decreases rapidly.

In the second stage (35–130 ms), these bubbles merge and generate an uneven vapor layer, and the cycle of shrinkage and regeneration occurs, exposing some parts of the electrode to saline. After 48 ms, the vapor layer becomes thinner and more stable, and, then, the strong electric field generated by the high voltage causes the vapor layer to break down. At the same time, the high gas temperature of the plasma can maintain the vapor-layer stability. Finally, a stable vapor layer forms on each electrode surface and a plasma glow is observed.

In the final third stage (after 130 ms), the plasma scalpel enters the stable discharge mode, all three electrodes discharge stably, and three separate vapor layers merge to a large vapor layer. As the discharge continues, the plasma heating enhances water evaporation and generates a large bubble near the electrode (over 2 s as can be seen in Figure 5) [8,15]. The above-mentioned accurate diagnostics of the plasma scalpel’s plasma characteristics enable us to improve its treatment efficiency more effectively.

## 4. Thermal Effect of Plasma Scalpel

The frequency of the plasma driving voltage is at least 100 kHz to avoid electrical stimulation of nerve and muscle cells at lower frequencies. The plasma scalpel’s thermal effect in tissue mainly depends on the heating rate and the temperature T reached, which, in turn, depends on the plasma power and treatment time [6]. The plasma scalpel effects on tissue can generally be divided into coagulation (T < 100 °C, slower heating) and cutting (T > 100 °C, faster heating) [31,32]. The higher heat required for the high-intensity heating required for cutting is generated by spark discharges between the electrodes and the tissue. Coagulation refers to tissue inactivation and hemostasis through cell drying and protein denaturation. The plasma’s discharge voltage can be adjusted to achieve the desired effects, for example, to control the degree of coagulation at the edges of surgical incisions [33,34].

Systematically grasping the biological tissue’s thermal effect can improve the plasma scalpel’s treatment effect more effectively. At temperatures below 40 °C, irreversible cellular damage does not usually occur in human tissue. When the temperature rises to 40–50 °C, the cell membrane and molecular structure change, and, at the same time, edema forms due to the transportation of water by the cells, and tissue fluid increases. Tissue fluid is extracellular fluid which bathes the cells of most tissues, arriving via blood capillaries and being removed via the lymphatic vessels. Depending on the duration of the heating, these changes are still reversible, but if the heating continues for a few minutes, the tissue begins to die. The higher temperatures damages cells irreversibly after a few seconds. When the temperature rises to 60–80 °C, cells containing a large amount of proteins is destroyed, since the proteins inside the cells are denatured, which leads to cell necrosis. The intercellular proteins are also coagulated and converted into gelatin. When the temperature rises to 100 °C, the cell fluid begins to evaporate; afterwards, the temperature rises above 100 °C, and the remaining material may burn and cause unwanted carbonization [6,17,33,35].

**Figure 6:**
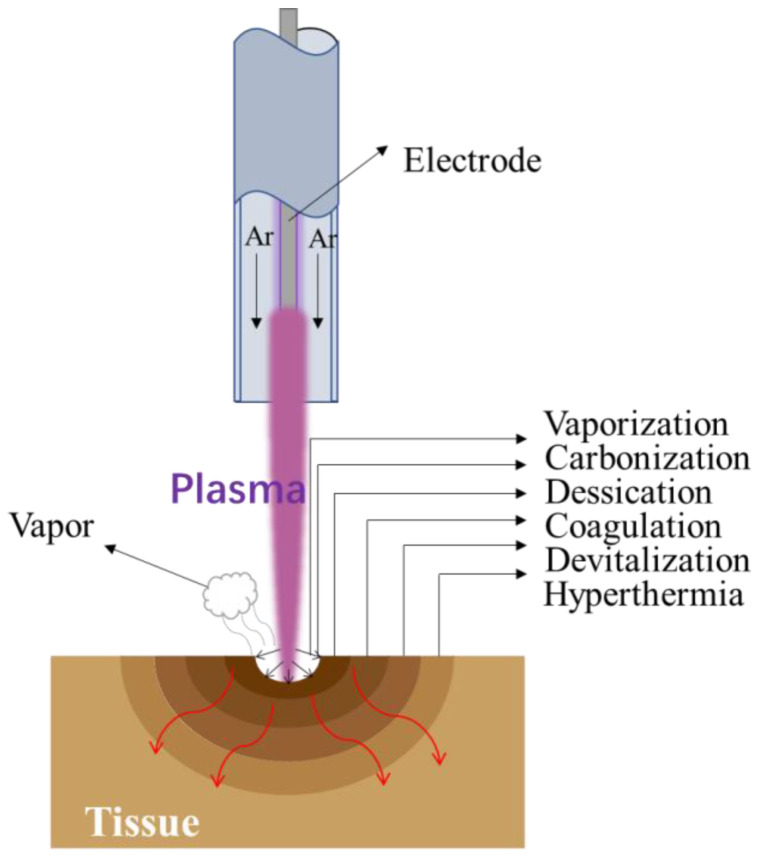
The schematic diagram of the thermal effect of the plasma scalpel.

The thermal effect of the plasma scalpel is shown in Figure 6. Depending on the distance from the plasma, evaporation, carbonization, dessication, coagulation, devitalization, and hyperthermia occur in sequence [33,35]. Plasma tissue cutting occurs when the plasma contacts the tissue and the temperature rapidly rises to 100 °C or higher, causing the cells to vaporize and explode. With pure cutting, thermal damage to the cut edges is minimal as heat is dissipated into the resulting vapor envelope. The underlying carbonized layer is thin enough to effectively control blood seepage from the original surface area. The temperature of the tissue below the carbonized layer is between 60–95 °C, which can produce dessication and coagulation. Dessication occurs due to the loss of cellular moisture through the affected cell membrane. Coagulation occurs due to thermal protein denaturation. This is helpful for wound recovery. Devitalization and hyperthermia occur in deeper tissues. When normal body temperature is raised no irreversible effects will occur until the tissue temperature reaches 41.5 °C. This temperature zone is called “hyperthermia”. If the temperature of human tissue is raised above 41.5 °C, an irreversible “devitalization” effect will occur. This has certain implications for the treatment of tumors [36,37].

## 5. Oxidation Effect of Plasma Scalpel

Simulations show that the discharge of the plasma scalpel in the water-vapor layer (50% H_2_O and 50% H_2_ vapor mixture) generates a large number of reactive species, with H atoms being the most abundant (approximately 400 ppm), followed by O atoms (20 ppm) and OH (3–10 ppm). The rate constant of the OH radical extraction of H is about 5000 times faster than that of H atom and 500 times faster than that of the ground state oxygen atom [4,38,39,40,41,42]. The experimental spectral data show that a large number of hydroxyl radicals generated by the plasma scalpel [4] can achieve oxidative etching of tissue [43,44,45,46].

**Figure 7:**
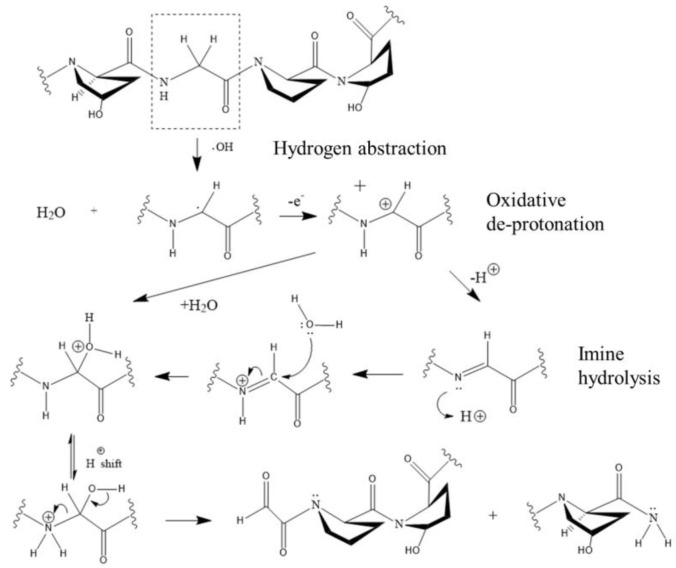
The chemical mechanism of the plasma scalpel inducing collagen fragmentation. Carbon-nitrogen cleavage occurs via OH extraction of hydrogen, hydroxylation of carbon, and hydrolytic cleavage of C-N bonds.

**Figure 8:**
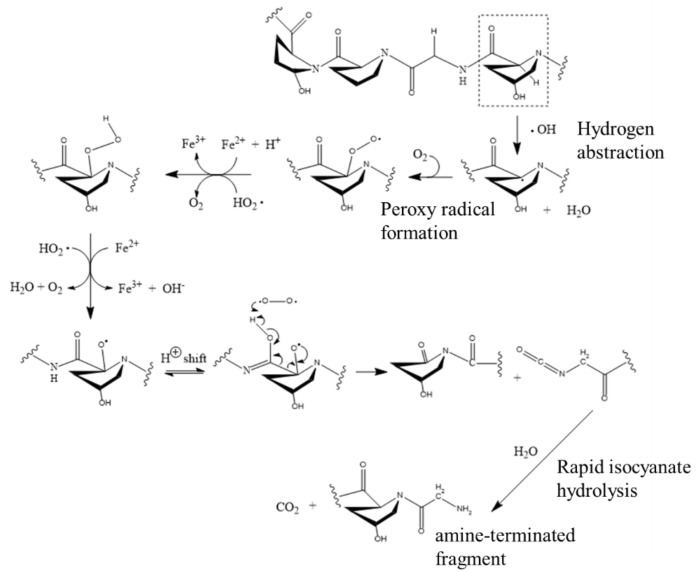
Chemical mechanism of collagen scission by the plasma scalpel through OH abstraction, hydroxylation of carbon, and oxidative cleavage of C-C bonds.

On the other hand, the protein collagen is the main fibrous element that comprises the matrix of skin, tendons, bones, cartilage, and teeth, and it consists of at least 20 different collagen types; for example, articular cartilage is composed of >90% collagen type II and small amounts of collagen types IX and XI; therefore, we analyze the key mechanism of the type II collagen fragmentation caused by the interaction with hydroxyl radicals and found that there are two possible pathways [4]. Figure 7 and Figure 8 show the degradation process of the hydroxyproline–glycine–proline–hydroxyproline moiety representing the polypeptide backbone after reacting with hydroxyl radicals. Figure 7 shows OH extracting H from glycine residues, resulting in cleavage of carbon–nitrogen bonds, while Figure 8 shows OH abstracting H from hydroxyproline residues, resulting in cleavage of carbon–carbon bonds. The common features of these two pathways include: 1. the initial removal of activated extracted hydrogen is due to the presence of adjacent nitrogens; 2. the resulting free radical center is located between the amide nitrogen and the carbonyl carbon, and this center is subsequently oxidized. In fact, the hydroxylation shown in Figure 7 leads to the activation of the structure to hydrolytic cleavage, and the result shown in Figure 8 is activated to oxidative cleavage; and 3. the hydroxylation of the initial radical site is induced by a radical reaction or a proton transfer reaction.

## 6. Theoretical Study on Plasma Scalpels

Theoretical research on plasma biomedicine can also improve the design of the plasma scalpel and expand its application [47]. The 2D simulation of discharge in water provides high spatial and temporal resolution of the dynamic process of plasma development, electric field, electron energy, and other physical information in water and biological tissue, which cannot be obtained by current experimental diagnosis [48,49]. These key parameters are of great significance for improving scalpel design and reducing patient discomfort during treatment. The distribution of the plasma-generated RONS, such as O, OH, and H_2_O_2_ in the liquid or biological tissue provided by the simulation, is also important information for researchers, especially for the molecular dynamics simulation which allows researchers to understand the interaction between plasma and cells at the atomic and molecular levels [50,51]. Understanding plasma’s bio-chemical effect is of great significance in expanding the role of plasma scalpels in cancer treatment.

## 7. Medical Applications of Plasma Scalpels

The plasma scalpel is suitable for almost all surgical fields, such as ear–nose–throat (ENT), gastroenterology, gynecology, urology, visceral surgery, etc. The main application areas are tissue cutting, ablation, and coagulation of tissue and blood vessels, emphasizing internal endoscopic surgical treatment.

### 7.1. Hemostasis

Bleeding can occur during surgery because of ulcers, injured or dilated blood vessels, and tumors. Plasma scalpels can treat relatively large surfaces quickly and efficiently, reducing blood loss, so that plasma scalpels have been used widely in tonsillectomy in otolaryngology [52], hemorrhagic cystitis treatment in urology [53], and large bowel hemorrhage due to radiation injury in gastrointestinal surgery [54,55].

### 7.2. Inactivation and Tissue Shrinkage

Treatment of tumors and occluded tissue requires inactivation and shrinkage. For early-stage tumors, one needs to kill the cancerous tissue and remove it mechanically. To treat incurable tumors and blockages caused by swollen tissue, we can use plasma scalpels to coagulate and dry them, thereby shrinking them; we can also further increase the discharge power to carbonize or even vaporize these tissues.

Typical applications of plasma scalpel inactivation and tissue reduction include minimally invasive treatment of tumors and metastases in the gastrointestinal and respiratory tracts, and tympanic membrane perforation repair surgery. In dermatologic treatments, the plasma scalpel can also be used for treating hemangiomas and warts. The advantages of the plasma scalpel include:

1. The connection between the lesion and the normal structure can be made clearer in the continuous perfusion mode;
2. The plasma scalpel head area temperature is about 40–70 °C, the operation temperature is low, and the bleeding can be reduced;
3. The advantages of underwater bone grinding during the operation of the plasma scalpel include effective washing of the operation cavity and further reduction of the temperature during the operation [56,57];
4. The lens of the plasma scalpel can be repositioned, realizing fine observation and precise surgery [4,58];
5. The function of the plasma scalpel head is integrated, which can realize surgical operations such as cutting, hemostasis, scraping, peeling, and pushing away, and shorten the operation time [8].

## 8. Conclusions and Outlook

This paper focused on the device structure and physicochemical parameters of two types of plasma scalpels, one is argon single electrode discharge technology, and the other is a two-electrode discharge device in normal saline. We also introduced the diagnostic method, including voltage and current characteristics, OES, ESR, and a high-speed camera, which can be used to obtain the key parameters, such as the plasma power, gas temperature, electron density, the density of active species, and the dynamics of the plasma discharge development in water. The response of biological tissue to the thermal effect and oxidation effect of plasma scalpels are systematically analyzed. The applications of plasma scalpels in the fields of hemostasis and tissue ablation are also discussed.

This paper reveals that the efficacy of the plasma scalpel is mainly based on the physical effects of the electric current and electric field, in addition to the chemical effects of high-density energetic electrons and RONS. The physical effects provided by the current and electric field can be further strengthened by optimizing the high-voltage electrode geometry and power driving frequency. This feature is helpful to improve the cutting and coagulation efficacy of the plasma scalpel, and to ensure that the coagulated tissue does not adhere to the electrode. The energy and density of electrons can also be increased by ramping the rising edge of the driving voltage pulse faster, thereby maintaining the chemical effect under the lower plasma temperature. Low-temperature operation is crucial for improving the treatment outcomes of the plasma scalpel for temperature-sensitive treatment objects.

In order to further promote the adoption and applications of plasma scalpel, the community should continue to: 1. improve the design of the electrode to enable it to treat tumor tissues with complex three-dimensional structures more effectively; 2. develop pulsed high-voltage power source with a faster-rising edge, enabling the plasma scalpel to generate higher density of active substances; 3. develop a high-precision simulation of the plasma breakdown process in the vapor layer; 4. study the microscopic mechanisms of the interaction between high-energy electrons passing through the vapor layer and biological tissues; and 5. systematically study the mechanisms of oxidation etching caused by the reactive species generated by plasma, such as H_2_O_2_, ^1^O_2_, HONOO, etc.

## Data Availability

The data that support the findings of this study are available from the corresponding author upon a reasonable request.

## References

[1] Morrison C.F. (1977). Electrosurgical Method and Apparatus for Initiating an Electrical Discharge in an Inert Gas Flow. U.S. Patent.

[2] Farin G., Grund K.E. (1994). Technology of Argon Plasma Coagulation with Particular Regard to Endoscopic Applications. Endosc. Surg. Allied Technol..

[3] Raiser J., Zenker M. (2006). Argon Plasma Coagulation for Open Surgical and Endoscopic Applications: State of the Art. J. Phys. D Appl. Phys..

[4] Stalder K.R., McMillen D.F., Woloszko J. (2005). Electrosurgical Plasmas. J. Phys. D Appl. Phys..

[5] Ly L., Jones S., Shashurin A., Zhuang T., Rowe W., Cheng X., Wigh S., Naab T., Keidar M., Canady J. (2018). A New Cold Plasma Jet: Performance Evaluation of Cold Plasma, Hybrid Plasma and Argon Plasma Coagulation. Plasma.

[6] Zenker M. (2008). Argon Plasma Coagulation. GMS Krankenh. Interdiszip..

[7] Folch E.E., Oberg C.L., Mehta A.C., Majid A., Keyes C., Fernandez-Bussy S. (2021). Argon Plasma Coagulation: Elucidation of the Mechanism of Gas Embolism. RES.

[8] Wu F., Li J., Liu F., Lu X. (2020). Ignition Phase of a Typical Plasma Scalpel. J. Phys. D Appl. Phys..

[9] Bürger I., Bibinov N., Neugebauer A., Enderle M., Awakowicz P. (2017). Electrical, Optical and Spectroscopic Characterisation of a Radio Frequency Discharge Used for Electrosurgical Cutting. Plasma Process. Polym..

[10] Korolev Y.D., Shemyakin I.A., Kasyanov V.S., Geyman V.G., Bolotov A.V., Nekhoroshev V.O. (2018). Development of Discharge in a Saline Solution at Near-Threshold Voltages. Plasma Phys. Rep..

[11] Korolev Y.D., Landl N.V., Bolotov A.V., Kasyanov V.S., Nekhoroshev V.O., Shemyakin I.A. (2022). Initial Stages of Pulsed Discharge in the Saline Solutions in a Vicinity of Threshold Voltages. Plasma Sources Sci. Technol..

[12] Hillebrand B., Iglesias E., Gibson A.R., Bibinov N., Neugebauer A., Enderle M., Awakowicz P. (2020). Determination of Plasma Parameters by Spectral Line Broadening in an Electrosurgical Argon Plasma. Plasma Sources Sci. Technol..

[13] Keller S., Bibinov N., Neugebauer A., Awakowicz P. (2012). Electrical and Spectroscopic Characterization of a Surgical Argon Plasma Discharge. J. Phys. D Appl. Phys..

[14] Weiss M., Utz R., Ackermann M., Taran F.-A., Krämer B., Hahn M., Wallwiener D., Brucker S., Haupt M., Barz J. (2019). Characterization of a Non-Thermally Operated Electrosurgical Argon Plasma Source by Electron Spin Resonance Spectroscopy. Plasma Processes Polym..

[15] Stalder K.R., Woloszko J. (2007). Some Physics and Chemistry of Electrosurgical Plasma Discharges. Contrib. Plasma Phys..

[16] Trimukhe A.M., Pandiyaraj K.N., Patekar M., Miller V., Deshmukh R.R. (2022). Perspectives and Advances of Nonthermal Plasma Technology in Cancers. IEEE Trans. Plasma Sci..

[17] Sato Y., Takayama T., Sagawa T., Hirakawa M., Ohnuma H., Miyanishi K., Sato T., Takimoto R., Kobune M., Okamoto K. (2011). Argon Plasma Coagulation Treatment of Hemorrhagic Radiation Proctopathy: The Optimal Settings for Application and Long-Term Outcome. Gastrointest. Endosc..

[18] Cao Y., Qu G., Li T., Jiang N., Wang T. (2018). Review on Reactive Species in Water Treatment Using Electrical Discharge Plasma: Formation, Measurement, Mechanisms and Mass Transfer. Plasma Sci. Technol..

[19] Woloszko J., Stalder K.R., Brown I.G. (2002). Plasma Characteristics of Repetitively-Pulsed Electrical Discharges in Saline Solutions Used for Surgical Procedures. IEEE Trans. Plasma Sci..

[20] Palanker D., Vankov A., Jayaraman P. (2008). On Mechanisms of Interaction in Electrosurgery. N. J. Phys..

[21] Verreycken T., van Gessel A.F.H., Pageau A., Bruggeman P. (2011). Validation of Gas Temperature Measurements by OES in an Atmospheric Air Glow Discharge with Water Electrode Using Rayleigh Scattering. Plasma Sources Sci. Technol..

[22] Staack D., Fridman A., Gutsol A., Gogotsi Y., Friedman G. (2008). Nanoscale Corona Discharge in Liquids, Enabling Nanosecond Optical Emission Spectroscopy. Angew. Chem..

[23] Huang Z., Xiao A., Liu D., Lu X., Ostrikov K. (2022). Plasma-Water-Based Nitrogen Fixation: Status, Mechanisms, and Opportunities. Plasma Processes Polym..

[24] Laroussi M., Bekeschus S., Keidar M., Bogaerts A., Fridman A., Lu X., Ostrikov K., Hori M., Stapelmann K., Miller V. (2022). Low-Temperature Plasma for Biology, Hygiene, and Medicine: Perspective and Roadmap. IEEE Trans. Radiat. Plasma Med. Sci..

[25] Chen B., Liu D. (2021). Mass Spectrometry Study on Ions Generated by Low-Temperature Plasma Jet. IEEE Trans. Plasma Sci..

[26] Lu X., Ostrikov K. (2018). Guided Ionization Waves: The Physics of Repeatability. Appl. Phys. Rev..

[27] Lu X., Keidar M., Laroussi M., Choi E., Szili E.J., Ostrikov K. (2019). Transcutaneous Plasma Stress: From Soft-Matter Models to Living Tissues. Mater. Sci. Eng. R Rep..

[28] Hofmann S., van Gessel A.F.H., Verreycken T., Bruggeman P. (2011). Power Dissipation, Gas Temperatures and Electron Densities of Cold Atmospheric Pressure Helium and Argon RF Plasma Jets. Plasma Sources Sci. Technol..

[29] Bruggeman P., Iza F., Guns P., Lauwers D., Kong M.G., Gonzalvo Y.A., Leys C., Schram D.C. (2009). Electronic Quenching of OH(A) by Water in Atmospheric Pressure Plasmas and Its Influence on the Gas Temperature Determination by OH(A–X) Emission. Plasma Sources Sci. Technol..

[30] Torres J., Palomares J.M., Sola A., van der Mullen J.J.A.M., Gamero A. (2007). A Stark Broadening Method to Determine Simultaneously the Electron Temperature and Density in High-Pressure Microwave Plasmas. J. Phys. D Appl. Phys..

[31] Qiuping Z., Cheng C., Yuedong M. (2009). Electron Density and Temperature Measurement by Stark Broadening in a Cold Argon Arc-Plasma Jet at Atmospheric Pressure. Plasma Sci. Technol..

[32] Lacitignola L., Desantis S., Izzo G., Staffieri F., Rossi R., Resta L., Crovace A. (2020). Comparative Morphological Effects of Cold-Blade, Electrosurgical, and Plasma Scalpels on Dog Skin. Vet. Sci..

[33] Rowe W., Cheng X., Ly L., Zhuang T., Basadonna G., Trink B., Keidar M., Canady J. (2018). The Canady Helios Cold Plasma Scalpel Significantly Decreases Viability in Malignant Solid Tumor Cells in a Dose-Dependent Manner. Plasma.

[34] Vargo J.J. (2004). Clinical Applications of the Argon Plasma Coagulator. Gastrointest. Endosc..

[35] Furtado F.S., Furtado G.B., Oliveira A.T., Oliveira F.A.A., Pinho C.S., Sampaio J.P.A., Feitosa A.M.L., de Lima Herculano Junir J.R. (2021). Endorectal Formalin Instillation or Argon Plasma Coagulation for Hemorrhagic Radiation Proctopathy Therapy: A Prospective and Randomized Clinical Trial. Gastrointest. Endosc..

[36] Jazrawi S.F., Nguyen D., Barnett C., Tang S. (2009). Novel Application of Intraductal Argon Plasma Coagulation in Biliary Papillomatosis (with Video). Gastrointest. Endosc..

[37] Overgaard K., Overgaard J. (1977). Hyperthermic Tumour-Cell Devitalization in Vivo. Acta Radiol. Ther. Phys. Biol..

[38] Schneider A., Feussner H., Schneider A., Feussner H. (2017). Chapter 6—Classical (Open) Surgery. Biomedical Engineering in Gastrointestinal Surgery.

[39] Zhang Y., Cheng H., Gao H., Liu D., Lu X. (2021). On the Charged Aerosols Generated by Atmospheric Pressure Non-Equilibrium Plasma. High Voltage.

[40] Yang Z., Liu D. (2021). Enhanced Transmembrane Transport of Reactive Oxygen Species by Electroporation Effect of Plasma. Plasma Processes Polym..

[41] Gao H., Li J., Liu D. (2021). Enhanced Aerosol Deposition by a Low-Cost Compact Nanosecond-Pulsed Plasma System. Plasma Processes Polym..

[42] Liu D., Zhang Y., Xu M., Chen H., Lu X., Ostrikov K. (2020). Cold Atmospheric Pressure Plasmas in Dermatology: Sources, Reactive Agents, and Therapeutic Effects. Plasma Processes Polym..

[43] Liu D., Szili E.J., Ostrikov K. (2020). Plasma Medicine: Opportunities for Nanotechnology in a Digital Age. Plasma Processes Polym..

[44] Lu X., Reuter S., Laroussi M., Liu D., Reuter S., Laroussi M., Liu D. (2019). Nonequilibrium Atmospheric Pressure Plasma Jets: Fundamentals, Diagnostics, and Medical Applications.

[45] Chu P.K., Lu X. (2013). Low Temperature Plasma Technology: Methods and Applications.

[46] Liu X.Y., Pei X.K., Ostrikov K., Lu X.P., Liu D.W. (2014). The Production Mechanisms of OH Radicals in a Pulsed Direct Current Plasma Jet. Phys. Plasmas.

[47] Liu X.Y., Pei X.K., Lu X.P., Liu D.W. (2014). Numerical and Experimental Study on a Pulsed-Dc Plasma Jet. Plasma Sources Sci. Technol..

[48] Vanraes P., Bogaerts A. (2018). Plasma Physics of Liquids—A Focused Review. Appl. Phys. Rev..

[49] Vanraes P., Bogaerts A. (2021). The Essential Role of the Plasma Sheath in Plasma–Liquid Interaction and Its Applications—A Perspective. J. Appl. Phys..

[50] Neyts E.C., Yusupov M., Verlackt C.C., Bogaerts A. (2014). Computer Simulations of Plasma–Biomolecule and Plasma–Tissue Interactions for a Better Insight in Plasma Medicine. J. Phys. D Appl. Phys..

[51] Bogaerts A., Yusupov M., der Paal J.V., Verlackt C.C.W., Neyts E.C. (2014). Reactive Molecular Dynamics Simulations for a Better Insight in Plasma Medicine. Plasma Processes Polym..

[52] Bergler W., Huber K., Hammerschmitt N., Hörmann K. (2001). Tonsillectomy With Argon Plasma Coagulation (APC): Evaluation of Pain and Hemorrhage. Laryngoscope.

[53] Quinlan D.M., Naslund M.J., Brendler C.B. (1992). Application of Argon Beam Coagulation in Urological Surgery. J. Urol..

[54] Szura M., Pasternak A. (2015). Upper Non-Variceal Gastrointestinal Bleeding—Review the Effectiveness of Endoscopic Hemostasis Methods. World J. Gastrointest. Endosc..

[55] Daga S. (2021). Liver Mobilisation during Recipient Hepatectomy Using Argon Plasma Coagulation—Tricks of Trade. HPB.

[56] Jones O., Cheng X., Murthy S.R.K., Ly L., Zhuang T., Basadonna G., Keidar M., Canady J. (2021). The Synergistic Effect of Canady Helios Cold Atmospheric Plasma and a FOLFIRINOX Regimen for the Treatment of Cholangiocarcinoma in Vitro. Sci. Rep..

[57] Clarkson D. (2016). Surgery: Plasma Cutting in Ophthalmology. Optician.

[58] Reitberger H.H., Czugala M., Chow C., Mohr A., Burkovski A., Gruenert A.K., Schoenebeck R., Fuchsluger T.A. (2018). Argon Cold Plasma—A Novel Tool to Treat Therapy-Resistant Corneal Infections. Am. J. Ophthalmol..

